# Minimally invasive management of pediatric osteoarticular infections

**DOI:** 10.3389/fped.2022.1017035

**Published:** 2022-11-11

**Authors:** Rosa María Alcobendas, Esmeralda Núñez, Cristina Calvo

**Affiliations:** ^1^ Pediatric Rheumatology Unit, Hospital Universitario La Paz, Madrid, Spain; ^2^ Pediatrics Department, Hospital Materno-Infantil, Málaga, Spain; ^3^ Pediatric Infectious Diseases Department, Hospital Universitario La Paz, Fundación IdiPaz, Madrid, Spain; ^4^ Translational Research Network in Pediatric Infectious Diseases (RITIP), Madrid, Spain; ^5^ CIBER Enfermedades Infecciosas (CIBERINFEC), Madrid, Spain

**Keywords:** osteomyelitis, oral, arthrocentesis, children, osteoarticular infections, treatment, septic arthritis (SA)

## Introduction

Osteoarticular infections (OAI) is an umbrella term for inflammation usually due to bacterial infection of bone and/or joints. The term OAI includes osteomyelitis (OM), septic arthritis (SA), septic osteoarthritis, spondylodiscitis (SD), pyogenic sacroiliitis (PSI), septic tenosynovitis, and chondritis. Acute OAI are defined as the diagnosis within 2 weeks after the onset of clinical manifestations in a previously uninfected location (1, 2).

Historically, *Staphylococcus aureus* has been described as the most prevalent microorganism involved in OAI in any age group (3). However, in recent years *Kingella kingae* has been described as the main causative agent of OAI in children aged 6–48 months (4). *K. kingae* OAI are usually characterized by a mild clinical presentation, minor increase in biological markers, and a better outcome than those caused by other bacteria, especially *S. aureus* (5). Children with *S. aureus* OAI tend to be older, have an associated fever, and a marked rise in acute phase reactants levels and white blood cell counts (5, 6). Likewise, methicillin-resistant *S. aureus* (MRSA) OAI has been related to severe purulent complications, increased probability of secondary-procedure, and a significantly higher admission rate to the intensive care unit (7, 8).

These different degrees of clinical presentation call into question whether it is time to rethink traditional recommendations and start treating the patient on an individualized basis. In fact, there is growing evidence of good outcomes in patients with primary hematogenous OAI treated with a minimally invasive approach consisting of stricter surgical indications and short courses or no intravenous therapy.

## Route of antibiotic therapy

Traditionally, children with acute OAI receive intravenous antibiotic therapy for several weeks, then switch to oral therapy. However, classical practice of a long duration of parenteral therapy is currently a source of controversy since this practice has been associated with prolonged hospitalization, high cost, and sometimes the need for central venous access (3). For this reason, some authors have suggested the possibility of reducing the duration of intravenous antibiotic therapy to only a few days, and then switching to oral therapy (9–11).

Recently, Peltola et al. conducted a prospective, randomized, and controlled study assessing 131 children aged 3 months to 15 years with culture positive OAI (11). The patients were randomly assigned to receive clindamycin or a first-generation cephalosporin for 20 or 30 days, including an intravenous phase for the first 2 to 4 days. Their conclusions were that most cases of pediatric OAI could be treated for only 20 days with a short initial period intravenously with large doses of a well-absorbed antimicrobial, including in infections caused by *S. aureus*.

Furthermore, in a systematic review and meta-analysis about short- vs. long-course antibiotics in pediatric and adult patients with osteomyelitis, no significant difference in the rate of treatment failure was found. This study showed that short-course antibiotics might be as effective as long-course antibiotics for patients with osteomyelitis, although inconsistent results were found in studies on vertebral osteomyelitis (12).

In addition, antibiotics with longer half-lives such as dalbavancin are also under study, predominantly in adult patients, to avoid or reduce admission time. Dalbavancin is a lipoglycopeptide with activity against a wide spectrum of gram-positive bacteria, including MRSA. Its pharmacokinetic profile includes a prolonged elimination half-life of 14.4 days and good penetration into bone and synovial fluid (13), offering potential for outpatient parenteral antibiotic therapy (14, 15). A two-dose regimen of weekly dalbavancin has proven to be effective for the treatment of osteomyelitis in adults in a randomized clinical trial (16). Also, a low rate of adverse events has been notified in a recent retrospective multicenter study, including two pediatric patients (17). So dalbavancin is currently considered an alternative for the treatment of osteomyelitis in adults. Its use must be based on risk-benefit considerations (18). However, data on its clinical efficacy in children are still limited.

Beyond reducing intravenous treatment time, in early 2022 Wald-Dickler et al. published the results of a revision of 20 randomized controlled trials comparing oral to intravenous therapy for blood and bone infection, with seven of them regarding osteomyelitis in 1,321 adult patients. *Staphylococcus aureus* and *Pseudomonas aeruginosa* were the most common monomicrobial organisms (19). They concluded that oral antibiotic therapy was at least as effective as IV, showing higher adverse event rates and decreased patient satisfaction in the IV groups. They suggest considering oral therapy in patients who meet certain criteria, including clinical and hemodynamical stability, good oral tolerance, availability of oral therapy, and no psychosocial or logistical reasons to prefer IV therapy.

Interestingly, very similar conclusions and selection criteria for exclusive oral therapy were described by our group in 2018 based on the results of the prospective study performed in an only pediatric population. The authors compared 25 outpatients who received just oral antibiotics with 228 hospitalized children who received initial IV treatment. The patients who received oral antibiotic since the diagnosis had good general condition, adequate oral intake, close control, and acceptance by legal guardians. All the OAI outpatients had a favorable outcome without sequelae (6).

In fact, most recently, the results of a nationwide multicenter registry from the Spanish Network of Osteoarticular Infections were published. In this study, 893 children who initially received intravenous antibiotics were compared (group 1) with 64 children who received exclusively oral therapy (group 2). Patients from group 2 were characterized as being younger, having a lower percentage of *Staphylococcus aureus*, and a higher proportion of *Kingella kingae* than in group 1, without showing any complications or clinical sequelae in this group. They concluded that an exclusively oral administration could be a safe option in selected patients with OAI clinically suggestive of *K. kingae* etiology and without risk factors for developing sequelae and complications. They propose low-risk criteria that should be met to be selected for this option of treatment (Table 1) (20).

**Table 1:** Proposed criteria for minimally invasive approach (all must be fulfilled)^a^ (20).

• Good general condition • Appropriate oral tolerance • No underlying disease • Age ≥6 months–<3 years • CRP ≤ 80 mg/L • ESR/CRP ratio ≥0,67^b^ • No history of injury, skin infections or recent surgery • No local complications at onset • No cervical spondylodiscitis • Possibility of attending daily check-ups at the outpatient department • Patient's legal guardians provided informed consent.

ESR, erythrocyte sedimentation rate; CRP, C reactive protein.

^a^ Individualize in case of hip arthritis.

^b^ ESR measured in mm/h. CRP measured in mg/L.

Oral treatment is usually well tolerated, and most compliance failures are related to intercurrent processes (poor oral tolerance or vomiting). In addition, it provides increased patient comfort and decreases the risk of nosocomial infection associated with prolonged intravenous therapy (21), so further studies are needed. In fact, a multi-center clinical trial called BEST (BonE and Joint Infections—Simplifying Treatment in Children Trial) is currently being carried out. The results of this trial will possibly shed light on this issue.

## Surgery

Together with antibiotics, surgery plays a key role in the treatment of acute osteomyelitis and septic arthritis in children (22). Surgery makes it possible to obtain biological samples that are useful for identifying the etiologic agent and then guiding the selection and duration of antimicrobial therapy (23).

### Acute osteomyelitis

Surgery in patients with OM has been described as able to alter the process of bone necrosis, to remove the demineralized bone, and to clean the surrounding soft tissue, thereby reducing the bacterial load (24).

Historically, early biopsy and debridement were recommended. However, there is little current high-quality evidence on which to base current surgical practice, and some retrospective case series have shown that up to 90% of patients with an early OM can be cured with conservative treatment, especially when antibiotics are initiated during the first days of the onset of symptoms (25, 26).

Although most cases of hematogenous osteomyelitis resolve with antimicrobial therapy alone, surgical intervention may be required in patients who do not respond to antibiotic treatment for the suspicion of an underlying complication, to control the focus of infection and preserve joint function.

Despite the fact that no clear indications have been established, general guidelines recommend surgical drainage for patients who present multifocal disease, who do not respond to antibiotics after 48–72 h, where ther is radiological evidence of a substantial pus collection, and sequestration as well as in patients with septic aspect at onset (1, 23, 27–30). Data regarding abscess size that mandates surgical drainage are very limited, although drainage of abscesses 2 cm or more in diameter has been suggested (23).

In current practice, the relative roles of bacterial virulence, host age, and immunity are unclear. More invasive surgery appears more common when bacteria have specific virulence genes, such as PVL. Acute osteoarticular infections caused by MRSA or *S. aureus* producing PVL often require more surgical sessions as these bacteria are associated with a more aggressive clinical course (3, 31, 32). However, most children with *K. kingae* OAI respond rapidly to conservative treatment with appropriate antibiotics and do not usually require invasive surgical procedures to achieve clinical improvement (33).

### Spondylodiscitis

In most of the children with SD, conservative treatment without surgery is usually sufficient. Currently, surgical management is indicated in case of vertebral instability, neurological signs, or failure of conservative treatment (34–36).

### Drainage technique in SA

Acute septic arthritis in children is considered an orthopedic emergency. Its treatment includes drainage of the joint to reduce the risk of complications such as avascular necrosis of the bone and permanent cartilage damage due to increased intra-articular pressure (37). However, the literature is scarce with respect to the optimal drainage technique in children with SA.

According to the guideline of the European Society for Paediatric Infectious Diseases (ESPID), SA in children should be treated with joint drainage by aspiration (arthrocentesis), arthroscopy, or arthrotomy followed by intravenous antibiotics (1).

Recently, Spaans and Donders have published three systematic reviews of the literature (including retrospective and prospective studies) on drainage techniques for septic hip, knee, and shoulder arthritis in children (38–40). These joints are important in the pediatric population since the hip and knee are the most commonly affected locations in children with SA and hip and shoulder have been described as the joints with greater prognosis interest due to the potential risk of avascular necrosis. Although it is inappropriate to draw firm conclusions from these reviews, they could help to better understand the possible role of each technique in children with SA. Globally, these systematic reviews show that both aspiration and arthrotomy can achieve good clinical results in the treatment of SA. However, there are some points of interest.

First, hip SA patients treated by arthrotomy required fewer additional drainage procedures in comparison with arthroscopy and arthrocentesis. Arthrocentesis of hip joint followed by a drain seemed to be associated with less likelihood of an additional arthrotomy than only with arthrocentesis without a drain (40).

Nevertheless, inferior clinical outcomes and more radiological sequelae were seen in hip SA patients treated with an arthrotomy as the first approach. Furthermore, most of the patients with hip SA treated initially with arthrocentesis who showed radiological changes had needed an additional arthrotomy as a second step (40).

Similarly, Smith et al. also found a higher percentage of damage to the glenohumeral joint in the arthrotomy group in shoulder SA patients, but the difference was not statistically significant (41). However, most of the radiological sequelae described in knee SA patients were seen in those who were treated with arthrocentesis but without irrigation (42).

Second, the time between onset of symptoms and treatment has been also described as a possible predictor of clinical and radiological outcomes in different studies, being poor in those with higher delay (43–46).

Thirdly, age and acute phase reactants have also been assessed. Failure of joint aspiration was evaluated by Tornero et al. who conducted a retrospective study that included 74 children with septic knee arthritis initially treated with needle joint aspiration. They found that arthrocentesis did not require additional drainage in any patient younger than one year old and in all patients between 1 and 3 years with a CRP < 20 mg/l (47), possibly related to *K. kingae*.

Finally, small or difficult-to-access joints such as sacroiliac, sternoclavicular, or interphalangeal are notoriously more difficult to aspirate, especially in children. For this reason, in these cases, a trial of medical management with antibiotics could be attempted (20, 48, 49). In patients in whom a sample from the site of infection cannot be obtained, detection of *K. kingae* DNA in oropharynx could point to etiology by this bacterium (50).

## Conclusion

This paper presents an up-to-date look at the approach and treatment of pediatric patients with primary hematogenous OAI. In the light of recent research, it appears that, in selected patients, minimally invasive treatment could be a safe and effective option, although further studies are needed.
