# Corrigendum: Minimally invasive management of pediatric osteoarticular infections

**DOI:** 10.3389/fped.2023.1149737

**Published:** 2023-02-10

**Authors:** Rosa María Alcobendas, Esmeralda Núñez, Cristina Calvo

**Affiliations:** ^1^ Pediatric Rheumatology Unit, Hospital Universitario La Paz, Madrid, Spain; ^2^ Pediatrics Department, Hospital Materno-Infantil, Málaga, Spain; ^3^ Pediatric Infectious Diseases Department, Hospital Universitario La Paz, Fundación IdiPaz, Madrid, Spain; ^4^ Translational Research Network in Pediatric Infectious Diseases (RITIP), Madrid, Spain; ^5^ CIBER Enfermedades Infecciosas (CIBERINFEC), Madrid, Spain

**Keywords:** osteomyelitis, oral, arthrocentesis, children, osteoarticular infections, treatment, septic arthritis (SA)

Corrigendum on Minimally invasive management of pediatric osteoarticular infections By Alcobendas RM, Núñez E and Calvo C. Front Pediatr. (2022) 10:1017035. doi: 10.3389/fped.2022.1017035

In the published article, there was an error regarding the affiliation for Cristina Calvo. Affiliation 3 should read: “Pediatric Infectious Diseases Department, Hospital Universitario La Paz, Fundación IdiPaz, Madrid, Spain” and they should also have the following affiliations: 4) “Translational Research Network in Pediatric Infectious Diseases (RITIP), Madrid, Spain” 5) “CIBER Enfermedades Infecciosas (CIBERINFEC), Madrid, Spain”

The authors apologize for this error and state that this does not change the scientific conclusions of the article in any way. The original article has been updated.

